# Assessing causal relationships between gut microbiota and psoriasis: evidence from two sample Mendelian randomization analysis

**DOI:** 10.1038/s41598-024-59603-5

**Published:** 2024-04-17

**Authors:** Yuan Li, Gaihe Chen, Xiaohuan Hu, Yunlei Bao, Chuyan Wu, Ni Zeng, Feng Jiang

**Affiliations:** 1 Department of Dermatology, The Fifth People’s Hospital of Hainan Province, Haikou, China; 2 Department of Neonatology, Obstetrics and Gynecology Hospital of Fudan University, Shanghai, China; 3 Department of Rehabilitation Medicine, The First Affiliated Hospital of Nanjing Medical University, Nanjing, 210029 China; 4 Department of Dermatology, Affiliated Hospital of Zunyi Medical University, Zunyi, 563003 China

**Keywords:** Gut microbiota, Mendelian randomization, Psoriasis, GWAS, Genetics, Computational biology and bioinformatics, Microbiology

## Abstract

Mounting data hints that the gut microbiota's role may be pivotal in understanding the emergence of psoriasis. However, discerning a direct causal link is yet elusive. In this exploration, we adopted a Mendelian randomization (MR) strategy to probe the prospective causal interplay between the gut's microbial landscape and the predisposition to psoriasis. Genetic markers acting as instrumental variables for gut microbiota were extrapolated from a genome-wide association study (GWAS) encompassing 18,340 individuals. A separate GWAS yielded summary data for psoriasis, which covered 337,159 patients and 433,201 control subjects. The primary analysis hinged on inverse variance weighting (IVW). Additional methods like the weighted median approach and MR-Egger regression were employed to validate the integrity of our findings. Intriguing correlations emerged between psoriasis risk and eight specific bacterial traits. To illustrate: Mollicutes presented an odds ratio (OR) of 1.003 with a 95% confidence interval (CI) spanning 1.001–1.005 (p = 0.016), while the family. Victivallaceae revealed an OR of 0.998 with CI values between 0.997 and 0.999 (p = 0.023). Eubacterium (coprostanoligenes group) revealed an OR of 0.997 with CI values between 0.994 and 0.999 (p = 0.027). Eubacterium (fissicatena group) revealed an OR of 0.997 with CI values between 0.996 and 0.999 (p = 0.005). Holdemania revealed an OR of 1.001 with CI values 1–1.003 (p = 0.034). Lachnospiraceae (NK4A136 group) revealed an OR of 0.997 with CI values between 0.995 and 0.999 (p = 0.046). Lactococcus revealed an OR of 0.998 with CI values between 0.996 and 0.999 (p = 0.008). Tenericutes revealed an OR of 1.003 with CI values between 1.001 and 1.006 (p = 0.016). Sensitivity analysis for these bacterial features yielded congruent outcomes, reinforcing statistically significant ties between the eight bacterial entities and psoriasis. This comprehensive probe underscores emerging evidence pointing towards a plausible causal nexus between diverse gut microbiota and the onset of psoriasis. It beckons further research to unravel the intricacies of how the gut's microbial constituents might sway psoriasis's pathogenesis.

## Introduction

Psoriasis is a chronic immune-mediated inflammatory skin disease triggered by various environmental and endogenous factors in genetically susceptible individuals^1^. Clinically, it typically presents as well-defined scaly erythematous plaques and, in rare cases, can lead to life-threatening generalized erythroderma^2^. The histologic features of psoriasis include hyperproliferation and hyperkeratosis of epidermal keratinocytes, dilated microvessels in the superficial dermis and an associated inflammatory response. With a better understanding of the pathophysiologic mechanisms involved, various treatments have been investigated. Previously, psoriasis was considered a proliferative skin disease, and treatment efforts were primarily focused on antiproliferative approaches^3^. However, more recent studies revealing elevated levels of interleukin-17 (IL-17) in psoriasis lesions have shifted the treatment focus towards helper T-cell 17 (Th17) cells^4^.

In recent years, research on the gut flora has intensified, providing increasing evidence of a link between the gut-skin axis. Dysbiosis in the gut can affect systemic immune function, leading to dysregulation of homeostasis and impaired skin function, which, in turn, may contribute to developing skin disorders. Growing evidence now suggests that in patients with psoriasis, the gut flora plays a role in establishing intestinal immunity^5^. On the other hand, severe intestinal malnutrition is observed in psoriasis patients, resulting in low diversity and altered relative abundance of specific intestinal flora^6^.

Although gut flora has been linked to psoriasis, the exact causal relationship remains uncertain. One statistical method that can help infer potential causality from observed associations is Mendelian randomization (MR) analysis^7^. MR capitalizes on genetic variants correlated with the target exposure, using these as instrumental variables to discern associations between the exposure proxy and the outcome^8^. The adoption of MR techniques to explore possible causal links between gut microbiota and disease susceptibility genes has recently surged^9–11^. This trend underscores the pressing imperative to scrutinize the plausible causal connection between gut microbial composition and psoriasis susceptibility.

In this study, we conducted a two-sample MR analysis based on pooled data from Genome-Wide Association Studies (GWAS). The purpose was to investigate the potential causal relationship between gut microbiota and psoriasis and identifying specific taxa of pathogenic bacteria involved in the condition.

## Materials and methods

### Outcome data sources

**Figure 1:**
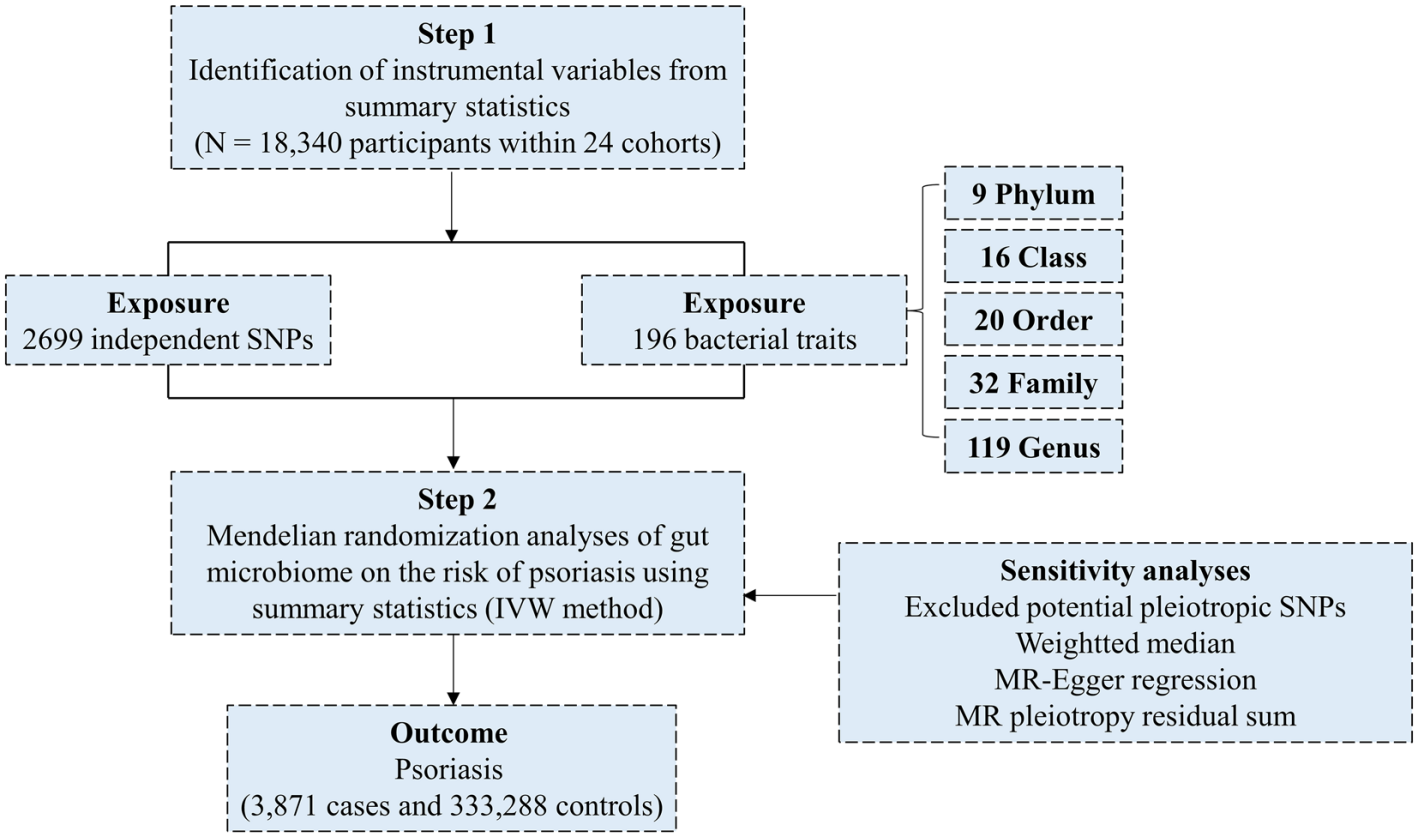
Study design of the MR analysis on the associations of gut microbiota and psoriasis. *GWAS* Genome Wide Association Studies, *MR* Mendelian randomization, *SNP* single nucleotide polymorphism.

Figure 1 offers a graphic depiction of this study's overarching design. Succinctly, we derived genetic summary metrics for psoriasis from a GWAS encompassing 337,159 cases and 433,201 controls, all of the European lineage. This GWAS amalgamates data sourced from both the UK Biobank.

To be included in the study, all patients with psoriasis were required to satisfy two criteria: item 5 and any of the first four items listed below: (1) Ordinary psoriasis: Characterized by erythematous, scaly lesions accompanied by punctate haemorrhages and the thin film phenomenon. (2) Psoriasis nail damage: Approximately 50% of psoriasis patients exhibit nail abnormalities, such as furrowed indentations, desquamation, and blotchy or oily changes. (3) Psoriatic arthritis: Approximately 30% of psoriatic arthritis patients also have psoriatic arthritis, which presents as joint pain, swelling, and movement limitations. (4) Hospital events with ICD-10 diagnostic codes: Statistical data on hospital events related to inpatient or day patient hospitalizations with a clinical diagnosis of psoriasis were considered using ICD-10 codes. (5) Histopathologic biopsy confirmation: The diagnosis of psoriasis was confirmed by histopathologic biopsy^12^.

This study utilized human gut microbiome summary statistics from the most recent GWAS meta-analysis, which integrated data from 18,340 participants spanning 24 distinct cohorts^13^. A more thorough delineation of this study can be found in another publication. To encapsulate, the research synchronized 16S rRNA gene sequencing patterns with genotyping data drawn from multiple international cohorts, including nations such as the United States, Canada, Germany, and others in Northern Europe. Association assessments were conducted, controlling for age, gender, technical covariates, and genetic principal components. Since our study relied on publicly accessible aggregated data, there was no mandate for further ethical clearance or participant consent. The particulars of the datasets employed in our MR exploration are itemized in Table 1.

**Table 1:** Details of the genome-wide association studies and datasets used in our analyses.

Exposure or outcome	Sample size	Ancestry	Links for data download	PMID
Human gut microbiome	18,340 participants	Mixed	https://mibiogen.gcc.rug.nl	33462485
Psoriasis	3871 cases, 333,288 controls	European ancestry	https://gwas.mrcieu.ac.uk/datasets/ukb-a-100/	35131400

### Selection of instrumental variables

Firstly, we removed 15 bacterial traits that did not have specific names, resulting in 196 remaining bacterial characteristics, which included nine species, 16 orders, 20 families, 32 genera, and 119 genera. Next, we selected instrumental variables (IVs) with a p < 1.0 × 10^–5^ significance level. To obtain site-independent IVs, we utilized the "TwoSampleMR" package and set the linkage disequilibrium (LD) threshold to R^2^ < 0.001. The clustering distance was also set to 10,000 kb, using 1000 genomic EUR data. We clustered the 196 bacterial traits, and for each cluster, we retained the SNP (single nucleotide polymorphism) with the lowest p-value among the associated traits. In total, we identified 2,699 independent SNPs that were associated with the 196 bacterial characteristics ^12^.

### Statistical analysis

This study assessed the potential causal relationship between gut microbiota and psoriasis using the fixed/random effects inverse variance weighted (IVW) method. The IVW method was chosen as the primary analysis due to its accurate effect estimation and its widespread utilization as the primary analysis in almost all MR studies^14–16^.

Incorporating many variants into MR analysis not only augments statistical potency but also risks ushering in pleiotropic genetic variants that could render instrumental variables questionable^17^. We implemented weighted median and MR-Egger regression strategies to navigate and offset pleiotropy. The MR-Egger regression stands resilient against dubious instruments. It recognizes unbalanced pleiotropy by instating a parameter designed to adjust for bias, capitalizing on summary data effect estimates drawn from multiple disparate variants^18^. The regression's gradient encapsulates the causal effect estimate, whereas the y-intercept offers insight into the overarching horizontal pleiotropic effect across the variants.

Meanwhile, the weighted median estimator yields a reliable causal effect projection, even when as much as half the input originates from potentially questionable genetic variants. Notably, this weighted median estimator upholds enhanced precision in its derivation. We pinned statistical significance at a P-value threshold below 0.05 across all tests in our investigation.

Correlations between the intricacies of human gut microbiota and the predisposition to psoriasis were quantified using odds ratios (ORs) bolstered by their corresponding 95% confidence intervals (CIs). The rigorous Bonferroni method was invoked to rectify multiple comparisons spanning diverse taxonomic echelons. Varied significance cut-offs were calibrated to the bacterial trait count within each gut microbiota tier for phylum, class, order, family, and genus. When p-values straddled between the demarcated significance bar and 0.05 signalled the potential for a causal association. All MR computations were orchestrated using R (version 3.6.3). To facilitate these evaluations, we deployed the "Mendelian Randomization" and "TwoSampleMR" packages, both of which are openly accessible.

### Heterogeneity and sensitivity test

To evaluate the heterogeneity among SNPs, we utilized Cochran's Q-statistics and I^2^ statistics^19,20^.

## Results

### The main results of the 196 bacterial traits with the risk of psoriasis

**Figure 2:**
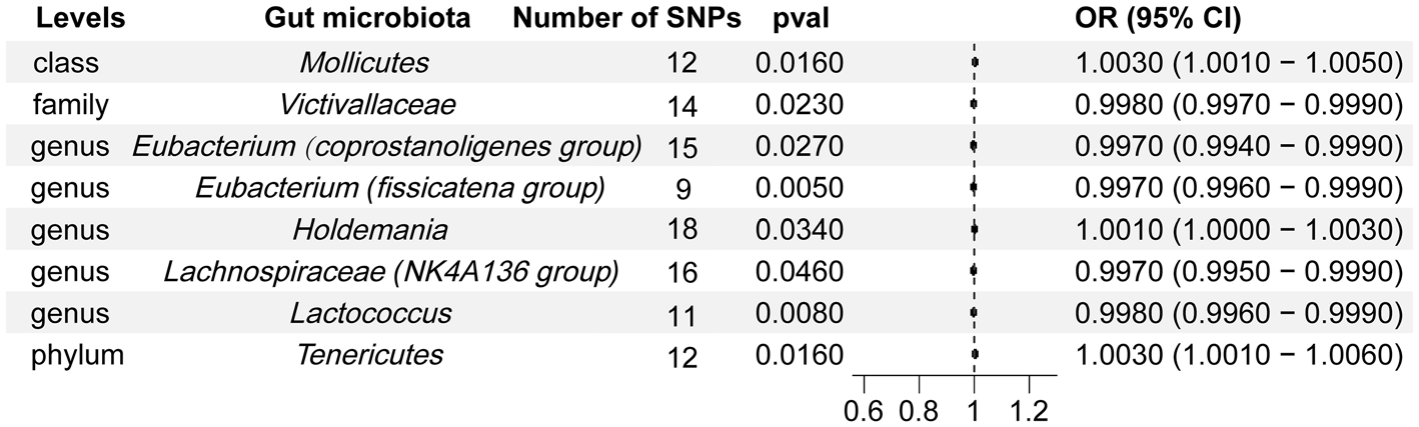
Forest plot of the associations between genetically determined 8 bacterial traits with the risk of psoriasis. The results is displayed in the form of odds ratio (OR) accompanied by their 95% confidence interval (CI). An odds ratio below 1 indicates a reduction in the risk of urolithiasis, while one above 1 indicates an increased likelihood of contributing to the development of the disease. *SNP* single nucleotide polymorphism.

**Figure 3:**
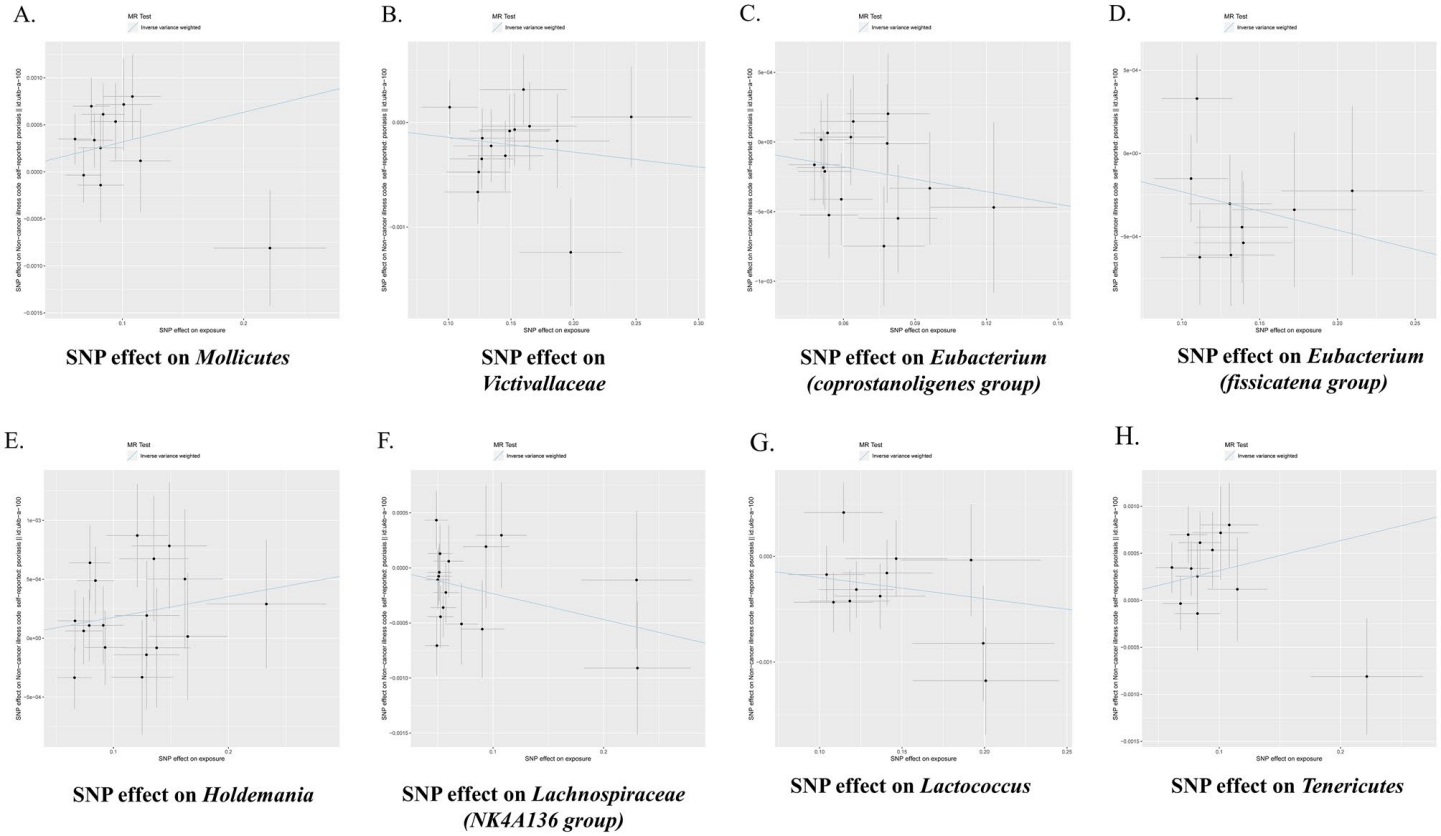
Scatter plots for the causal association between gut microbiota and psoriasis. The scatter plot shows that Mollicutes (**A**), Holdemania (**E**), Tenericutes (**H**) are positively correlated with the risk of psoriasis, while genus Victiallaceae (**B**), Eubacterium (coprostanoligenes group) (**C**), Eubacterium (fissicatena group) (**D**), Lachnospiraceae (NK4A136 group) (**F**) and Lactococcus (**G**) are negatively correlated with the risk of psoriasis. *SNP* single nucleotide polymorphism, *MR* Mendelian randomization.

**Figure 4:**
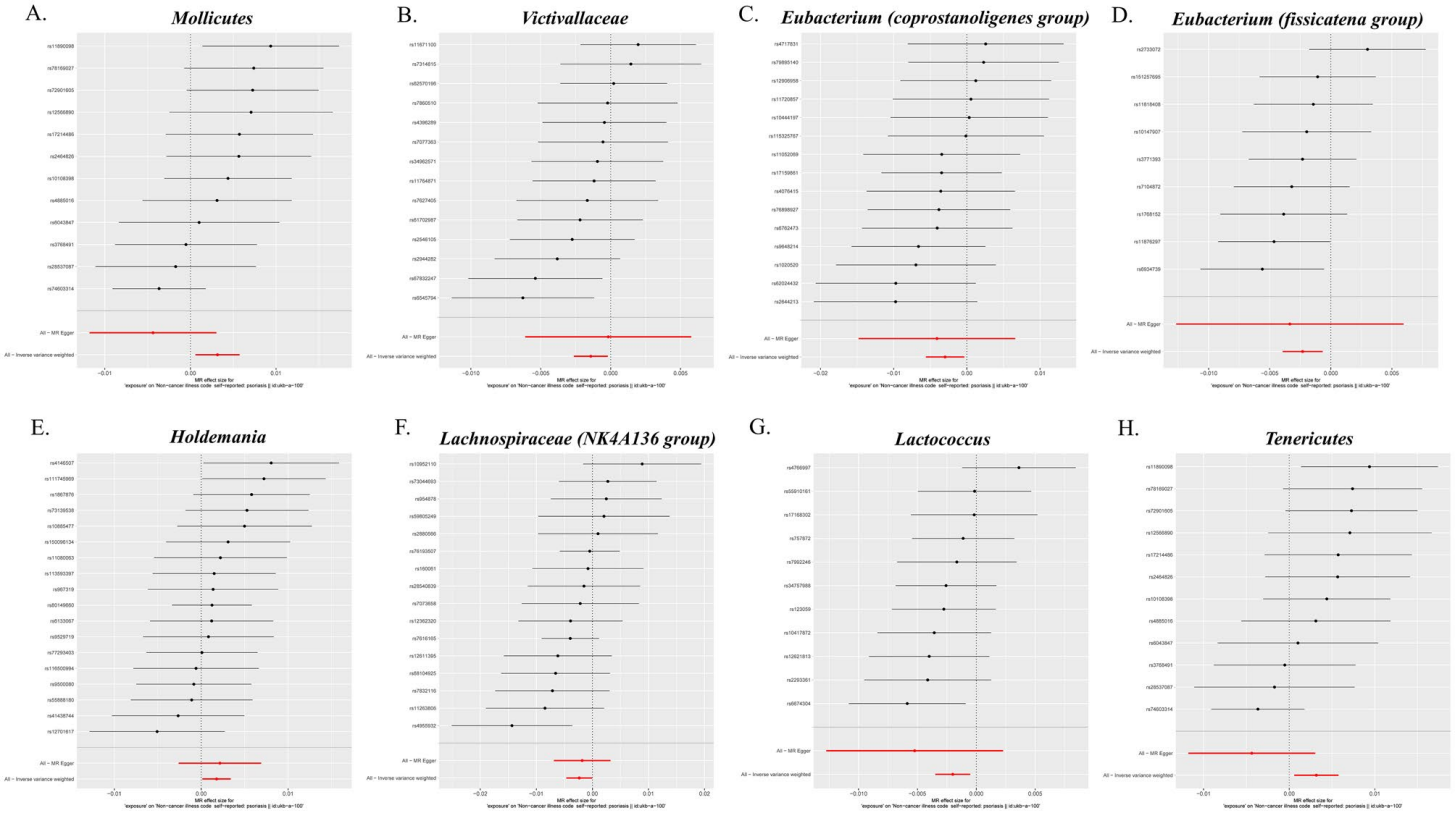
Leave-one-out plots show that the causal relationship between Mollicutes (**A**), genus Victiallaceae (**B**), Eubacterium (coprostanoligenes group) (**C**), Eubacterium (fissicatena group) (**D**), Holdemania (**E**), Lachnospiraceae (NK4A136 group) (**F**), Lactococcus (**G**) and Tenericutes (**H**) was not driven by any single SNP. *SNP* single nucleotide polymorphism, *MR* Mendelian randomization.

**Figure 5:**
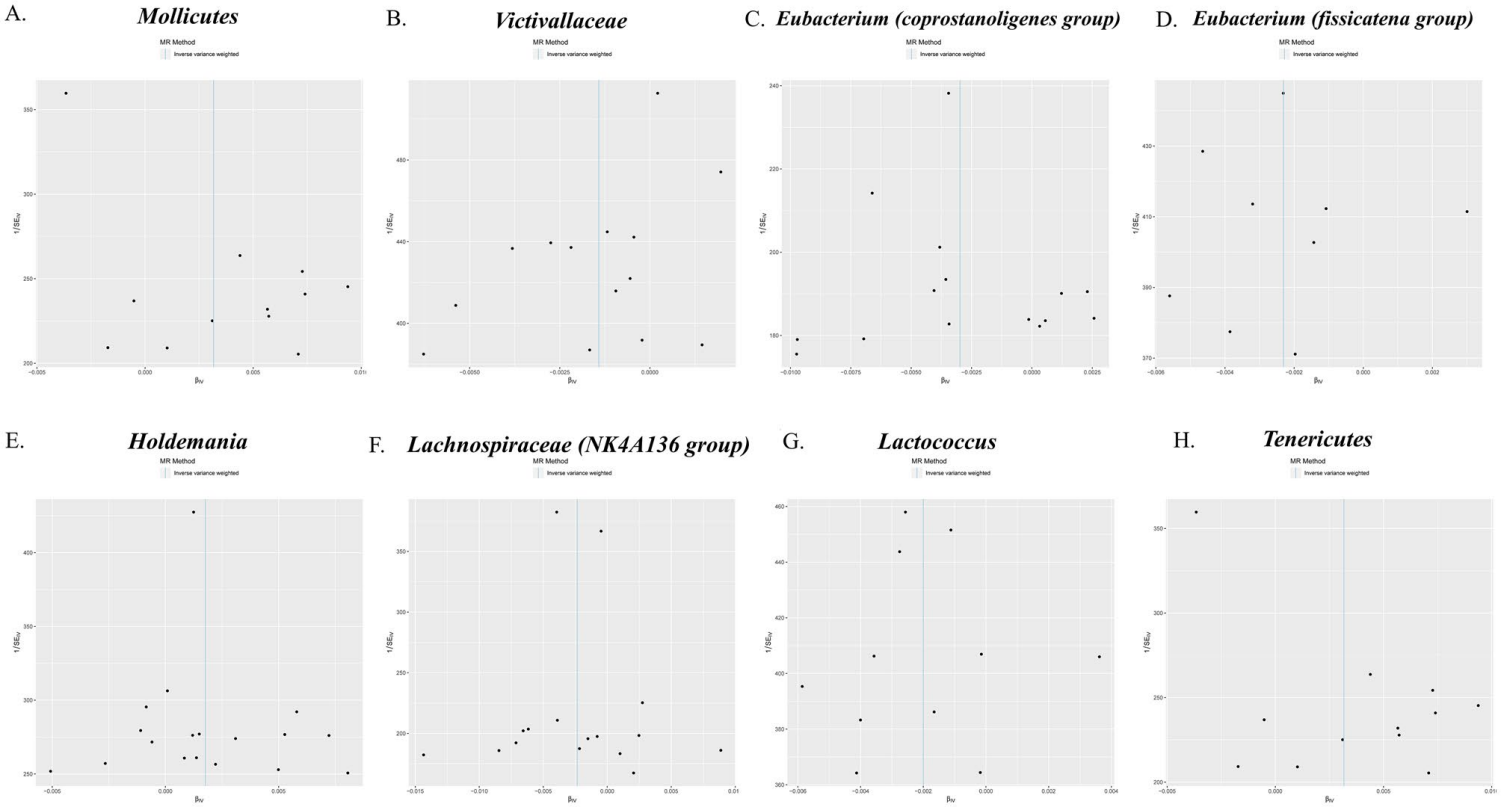
Funnel plot to assess heterogeneity. The blue line represents the inverse‐variance weighted estimate, and the dark blue line represents the Mendelian randomization‐Egger estimate.

The f-statistics for the 196 bacterial traits were all greater than 10, indicating a low likelihood of weak instrumental bias. notably, using the IVW method (Fig. 2), we observed suggestive evidence of an association between 8 bacterial characteristics and psoriasis risk. Characteristics of the genetic variants associated with eight bacterial that have been identified to be related to the risk of psoriasis can be found in Table S1. The IVs employed for these eight bacterial characteristics can be found in Table 2. In our study, we made several significant findings regarding the association between specific bacterial characteristics and the risk of psoriasis: using the IVW method, genetic predictions highlighted that mollicutes positively correlated with the risk of psoriasis (OR: 1.003; 95% CI: 1.001–1.005; p = 0.016) (See Fig. 3). The weighted median approach corroborated these findings (OR: 1.004; 95% CI 1.0007–1.007; p = 0.016). The MR-Egger regression analysis showed no inclination towards a directional pleiotropic influence (intercept p-value = 0.016) (Refer to Fig. 4), while the funnel plot demonstrated symmetry (Fig. 5).

**Table 2:** MR estimates from inverse variance weighted method of assessing the causal effect of gut microbiota on the risk of psoriasis.

Gut microbiota	Cochran Q statistic	I^2^	Heterogeneity P-value
Mollicutes	13.91	0.2092	0.238
Victivallaceae	12.80	0.0156	0.463
Eubacterium (coprostanoligenes group)	7.831	0.7877	0.897
Eubacterium (fissicatena group)	8.286	0.0345	0.406
Holdemania	14.217	0.1957	0.651
Lachnospiraceae (NK4A136 group)	16.843	0.1094	0.328
Lactococcus	10.496	0.0472	0.398
Tenericutes	13.909	0.2091	0.238

^a^I^2^ = (Q − d*f*)/Q.

Similarly, Holdemania positively correlated with psoriasis susceptibility when assessed via the IVW approach (OR: 1.001; 95% CI 1.000–1.003; p = 0.034) (Refer to Fig. 3). Consistent outcomes were gleaned from the weighted median method (OR: 1.001; 95% CI 0.998–1.003; p = 0.033). The MR-Egger regression further validated the absence of horizontal pleiotropy (intercept p-value = 0.033) (See Fig. 4), with the funnel plot maintaining symmetry (Fig. 5).

Tenericutes, as discerned from the IVW technique, showcased a positive tilt with the psoriasis risk profile (OR: 1.003; 95% CI 1.001–1.006; p = 0.016) (Refer to Fig. 3). Sensitivity analyses via the weighted median method mirrored these observations (OR: 1.00; 95% CI 0.98–1.007; p = 0.007). Moreover, MR-Egger regression presented scant evidence of a directional pleiotropic thrust (intercept p-value = 0.012) (See Fig. 4), with funnel plots maintaining their unbiased stance (Fig. 5).

Contrastingly, negative correlations between five bacterial attributes and psoriasis susceptibility emerged from the IVW method, notably for the following taxa: Victivallaceae, Eubacterium (coprostanoligenes group), Eubacterium (fissicatena group), another unidentified Genus, and Lactococcus (details provided in the original text) (See Fig. 3). Nonetheless, it's pertinent to highlight that the weighted median approach offered a more ambivalent stance on these perceived causal associations.

## Discussion

Psoriasis, also known as psoriasis vulgaris, is a chronic autoimmune disease characterized by red patches of skin covered with silvery-white scales. In 2019, there were 4,622,594 cases of psoriasis globally (95% uncertainty interval or UI 4,458,904–4,780,771). The incidence rate of psoriasis in 2019 was 57.8 cases per 100,000 individuals (95% UI 55.8–59.7). This represents a decrease of 20.0% (95% UI − 20.2 to − 19.8) compared to 1990. Stratifying by gender, the incidence rates for males [57.8 (95% UI 55.8–59.8) per 100,000 individuals] and females [57.8 (95% UI 55.8–59.7) per 100,000 individuals] were similar. Compared to 1990, the incidence rates decreased by 19.5% (95% UI − 19.8 to − 19.2) for males and 20.4% (95% UI − 20.7 to − 20.2) for females. It was observed that the incidence rate of psoriasis varied geographically, with the highest rates reported in high-income countries and regions [112.6 (95% UI 108.9–116.1)], followed by middle to high Sociodemographic Index (SDI) countries [69.4 (95% UI 67.1–71.9)], while the lowest rates were reported in low SDI countries [38.1 (95% UI 36.8–39.5)]. Similar trends were found regarding prevalence and disability rates^21^.

While primarily affecting the skin, psoriasis can also lead to various other health issues, often called comorbidities. Among the comorbidities associated with psoriasis are arthritis, cardiovascular diseases, metabolic syndrome, mental health issues, infections, kidney diseases, osteoporosis, and other autoimmune diseases such as rheumatoid arthritis or systemic lupus erythematosus^22^. Research indicates that psoriasis (PsO) patients are more likely to be diagnosed with 2–4 comorbidities (28.8% vs 23.8%) and > 5 comorbidities (19.6% vs 12.9%). The specific comorbidity profile of PsO patients reflects several core pathological processes, including autoimmune and systemic inflammatory diseases such as hidradenitis suppurativa (OR 3.55, 95% CI 1.88–7.28) or rheumatoid arthritis (OR 3.01, 95% CI 1.96–4.77), inflammatory bowel disease such as Crohn's disease (OR 2.99, 95% CI 2.20–4.13), pulmonary diseases such as chronic obstructive pulmonary disease (OR 1.81, 95% CI 1.61–2.04), hepatic diseases such as cirrhosis (OR 2.00, 95% CI 1.36–3.00), endocrine disorders such as thyroid dysfunction (OR 1.82, 95% CI 1.30–2.59), psychiatric disorders such as depression (OR 1.72, 95% CI 1.57–1.87), and cardiovascular diseases such as hypertension (OR 1.47, 95% CI 1.41–1.53)^23^. Dysbiosis of the gut microbiota can also result in several comorbidities, some of which may be related to the pathogenesis of psoriasis, thereby triggering or exacerbating the development of psoriasis. Firstly, dysbiosis of the gut microbiota may lead to immune system abnormalities. The gut microbiota is closely linked to the immune system, and dysregulated microbiota may cause either overactivation or diminished function of the immune system, increasing the risk of autoimmune diseases.

Psoriasis is an immune-mediated disease, and its pathogenesis is closely associated with immune system abnormalities, particularly abnormal activation of T cells^24^. Therefore, dysbiosis of the gut microbiota may increase the risk of psoriasis occurrence and development by influencing the immune system. Secondly, dysbiosis of the gut microbiota may result in increased inflammatory responses. Some studies indicate that dysbiosis of the gut microbiota may lead to damage and inflammation of the intestinal mucosa, releasing inflammatory mediators such as TNF-α, IL-6, etc. The excessive release of these inflammatory mediators may exacerbate the inflammatory response of psoriasis, promoting abnormal proliferation of skin cells and inflammatory reactions^25^.

Additionally, dysbiosis of the gut microbiota may also affect nutrient absorption and metabolism. The onset of psoriasis is associated with nutritional deficiencies or metabolic abnormalities. Dysregulated microbiota may lead to inadequate absorption of nutrients or the production of metabolites such as short-chain fatty acids, thereby affecting skin health and the development of psoriasis^26^.

Diet plays a crucial role in regulating patients' onset and progression of psoriasis. Although the etiology of psoriasis is complex, some studies suggest that adopting appropriate dietary habits can help alleviate symptoms, slow disease progression, and improve patients' quality of life. Certain foods contain anti-inflammatory components, such as fish, which are rich in Omega-3 fatty acids, nuts, seeds, fruits, and vegetables, as well as vitamin C, vitamin E, and other antioxidants^26^. These foods may help reduce inflammation and alleviate symptoms in psoriasis patients. Obesity is associated with the onset and severity of psoriasis. Therefore, maintaining a healthy weight and adopting proper dietary habits can reduce the release of inflammatory factors in the body, thus helping to alleviate psoriasis symptoms. Some psoriasis patients may be allergic or sensitive to certain foods, such as spicy foods, alcohol, caffeine, and dairy products. Avoiding or limiting these foods in personalized cases may help alleviate symptoms. Antioxidants help eliminate free radicals and reduce inflammatory responses. Increasing the intake of foods rich in antioxidants, such as fruits, vegetables, and tea, may help alleviate symptoms in psoriasis patients. Fluctuations in blood sugar levels may affect the immune system and inflammatory responses. Therefore, choosing foods with a low glycemic index (GI), such as whole grains, legumes, and vegetables, can help maintain stable blood sugar levels^27^. Deficiency in specific vitamins and minerals, such as vitamin D, vitamin A, zinc, and selenium, is associated with the occurrence and development of psoriasis. Ensuring an adequate intake of these nutrients may help alleviate symptoms^28^.

Systemic inflammation plays a significant role in regulating the onset and progression of psoriasis in patients. Psoriasis is a chronic inflammatory skin disease, and its pathogenesis is closely associated with immune system abnormalities, inflammatory responses, and abnormal proliferation of skin cells. Systemic inflammation can affect the regulation of the immune system, leading to excessive or aberrant activation of immune cells, thereby exacerbating the inflammatory response in psoriasis^29^. Inflammatory mediators such as tumor necrosis factor-alpha (TNF-α) and interleukin-17 (IL-17), among others, may play a crucial role in this process. Systemic inflammation can result in increased release of inflammatory mediators in the body, such as TNF-α and IL-17 mentioned above, which directly participate in the pathogenesis of psoriasis. They promote abnormal proliferation of keratinocytes and skin inflammation, thereby worsening the condition of psoriasis. Systemic inflammation may affect inflammatory signaling pathways in psoriasis patients, such as the nuclear factor-kappa B (NF-κB) signaling pathway, the Toll-like receptor (TLR) signaling pathway, etc. The signalling pathways abnormal activation or inhibition is closely associated with the onset and development of psoriasis. Systemic inflammation may affect the function of various immune cells, such as T cells, dendritic cells, etc., thereby regulating the activity of the immune system and affecting the immune-inflammatory response in psoriasis^30^.

Interleukin-17 (IL-17) and Interleukin-23 (IL-23) are crucial immunoregulatory factors in modulating the immune system's functions. They play pivotal roles in numerous immune responses, including combating infections and autoimmune diseases. IL-17, produced by T cells, particularly a subset known as Th17 cells, is capable of inducing inflammatory responses and plays a significant role in developing autoimmune diseases. IL-23, on the other hand, acts as a stimulant for the differentiation and proliferation of Th17 cells while regulating other different types of immune cells. The antagonistic effect of IL-17 and IL-23 pathways is crucial, particularly in infections such as intestinal nematode infection. Intestinal nematode infections trigger the activation of the host immune system, including the IL-17 and IL-23 pathways. IL-17 may promote inflammatory responses leading to tissue damage, but may also play a role in parasite clearance.

Conversely, IL-23 may play a crucial role in regulating inflammatory responses, but overactivation of the IL-23 pathway could lead to the development of inflammatory diseases. Hence, employing precision medicine approaches to avoid drug ineffectiveness is crucial. Precision medicine entails tailoring treatment plans based on individual characteristics such as genomic profiles, phenotypes, environment, and lifestyle. When dealing with the antagonistic effects of IL-17 and IL-23 pathways, precision medicine can aid in determining the patient's immune status, genetic background, and potential drug responses, thereby selecting the most appropriate treatment strategies^31^. This may involve selecting specific drug targets, adjusting dosages, or using combination therapies to minimize inflammatory responses, control infections, and reduce drug side effects and resistance risks. In conclusion, IL-17 and IL-23 pathways play critical roles in diseases such as intestinal nematode infection, and understanding and managing the interactions of these pathways are crucial for treatment success.

Both drug- and food-induced photosensitivity can potentially impact the treatment and condition of psoriasis. Firstly, drug-induced photosensitivity refers to adverse reactions that certain medications may cause when the skin is exposed to sunlight. Some medicines used to treat psoriasis patients may increase their sensitivity to sunlight, leading to photosensitivity reactions. For example, the use of photosensitizing drugs such as psoralen plus ultraviolet A (PUVA) therapy or certain oral medications (such as methoxsalen and acitretin) may increase patients' sensitivity to ultraviolet light, resulting in skin burns or other adverse reactions^32^. Secondly, food-induced photosensitivity refers to interactions between certain foods and sunlight that trigger skin reactions. Some foods' chemical components may increase the skin's sensitivity to ultraviolet light, leading to photosensitivity reactions. Some foods that may induce food-induced photosensitivity in psoriasis patients include celery, lemon, cilantro, fennel, and bergamot oil. Ingredients in these foods may interact with sunlight, causing skin allergic reactions or exacerbating the condition^33^.

Therefore, when treating psoriasis, doctors typically advise patients to avoid using photosensitizing drugs, especially during periods of intense sunlight, to reduce the risk of photosensitivity reactions. Patients should also be cautious about consuming foods that may induce food-induced photosensitivity, particularly during sun exposure. Considering both drug-induced photosensitivity and food-induced photosensitivity can help psoriasis patients manage their condition more effectively and reduce the occurrence of adverse reactions. Precision medicine approaches can optimize treatment outcomes and prevent drug ineffectiveness. Therefore, research suggests that the widespread use of artificial neural networks may help predict the efficacy of dermatological medications, providing valuable theoretical and clinical insights^34^.

In this study, we identified eight bacterial taxa with potential associations to the risk of psoriasis. These taxa include Mollicutes, Victivallaceae, Eubacterium (coprostanoligenes group), Eubacterium (fissicatena group), Holdemania, Lachnospiraceae (NK4A136 group), Lactococcus, Tenericutes. Our susceptibility analyses, conducted using different MR methods and restricted IV sets, suggest that these bacterial taxa may be linked to psoriasis risk. Further investigations and validation studies are warranted to confirm and understand the biological implications of these potential associations.

The human intestinal flora constitutes a complex micro-ecosystem comprising approximately 1000 microbial species and 1014 microorganisms. The number of microorganisms gradually increases from the stomach to the end of the colon^35^. The average human intestinal flora primarily consists of two main bacterial species: the thick-walled phylum and the anaplasma phylum^36^. Acknowledging that the human gut flora exhibits considerable variation among individuals is vital. Under homeostatic conditions, these bacteria play essential roles in digestion, metabolism, immune defence, and tolerance. A healthy and balanced intestinal flora is essential for maintaining these functions^37–39^.

Given the importance of gut flora, alterations in its composition have been associated with the pathogenesis of numerous acute and chronic diseases, such as diabetes, obesity, inflammatory bowel disease (IBD), Crohn's disease, autism, Alzheimer's disease, Parkinson's disease, anxiety, and depression^40–43^. For instance, studies have shown that 7–11% of patients diagnosed with IBD also have psoriasis, primarily linked to gastrointestinal inflammation^44^. Both diseases share certain common genetic and environmental factors and immune pathways. Notably, Th17 cells and their cytokines are known to play a significant role in developing psoriasis and are involved in the pathophysiology of IBD^45^. In the pathogenesis of both obesity and psoriasis, inflammation and adipokines play crucial roles, and a mutual promotion exists between the two conditions^46^. Numerous studies have demonstrated that obesity serves as an independent risk factor for psoriasis. It leads to an imbalance in the expression of pro- and anti-inflammatory adipokines in adipose tissue. It subsequently encourages the release of inflammatory cells and factors associated with psoriasis, thus contributing to cutaneous inflammation in psoriasis.

In addition, research has shown that a high-fat diet (HFD) lasting just one week can bring about significant changes in the fecal metabolome and gut microbiome of rats, and this alteration can persist for two months^47^. Analyses of the gut microbiome in HFD-induced obese mice have revealed the vigorous growth of a single evolutionary clade in the Mollicutes of Firmicutes (HFD). This study has highlighted a potential causal relationship between Mollicutes and psoriasis, underscoring the significance of Mollicutes in developing psoriasis^47^.

In a study conducted in China with 350 patients with psoriasis, it was observed that high-frequency alcohol consumption, including various types of alcoholic beverages such as white wine, red wine, and beer, as well as single large amounts of alcohol consumption, are associated with an increased risk of developing psoriasis. Ethanol, found in alcoholic drinks, plays a dual role in promoting psoriasis.

On one hand, ethanol can induce the release of pro-inflammatory cytokines from various cell types, either directly or through intermediate pathways. This can result in sustained systemic inflammation and facilitate the proliferation of lymphocytes^48^. This mechanism elucidates the potential contributory role of ethanol in inflammatory processes and related conditions. On the other hand, ethanol also influences the growth of intestinal flora. In populations that consume excessive amounts of ethanol, there is a higher relative abundance of Holdemania spp. in the gut microbiome. This, in turn, contributes to and exacerbates psoriasis^49^. These findings are consistent with the results of our study, which suggests a positive association between high levels of Holdemania and the risk of psoriasis.

Hyperglycemia has been found to impact intestinal epithelial cells, disrupting the intestinal vascular barrier and subsequent development of intestinal inflammation. In type 2 diabetic mice, this condition leads to specific changes in the gut microbiome. Notably, there is an increase in the abundance of Micrococcus warty and Tenericutes (soft-walled bacteria). In contrast, Mycobacterium avium and Sclerotium sclerotiorum (Sclerotium) bacteria experience a decrease in their levels compared to healthy mice^50^. However, it is essential to note that despite the positive association between Tenericutes and psoriasis risk observed in this study, there is currently no clear research evidence directly linking Tenericutes to psoriasis. Therefore, further investigations are required to explore the underlying biological mechanisms responsible for this potential association between Tenericutes and psoriasis.

The Eubacterium (coprostanoligenes group) and Eubacterium (fissicatena group) are intestinal microorganisms, consisting of several species of Eubacterium^36^. Eubacterium is an essential bacterium found in the colon of healthy individuals and is considered one of the core genera of the human gut^51^. It widely colonizes the intestinal tract, oral cavity, and other parts of the population, playing a vital role in nutrient metabolism and maintaining intestinal homeostasis^52^. Certain strains within the Eubacterium (coprostanoligenes group) possess steroid-metabolizing abilities, participating in cholesterol metabolism and converting cholesterol into the cholesterol degradation product, coprostanol^53^. This metabolic process is crucial for maintaining cholesterol homeostasis and normal cholesterol metabolism.

Members of the Eubacterium family are pivotal due to their capability to produce short-chain fatty acids, notably butyric acid^54^. These short-chain fatty acids have multifaceted roles in human health. For instance, they furnish specific nutrients and energy to the intestinal lining, fortify the intestinal mucosal barrier, temper inflammation, and augment gastrointestinal movement^55^. Various Eubacterium species generate butyrate, instrumental in energy balance, regulating colonic movements, modulating immune responses, and curtailing intestinal inflammation^56^. Butyrate, a short-chain fatty acid, is a fermentation product of Eubacterium spp. It can upregulate mucin twogene expression by directly acting on cuprates and may induce mucin two secretions through the intermediate product, prostaglandin^57^. Mucin 2 is the primary active component of the colorectal mucosal barrier, and apart from its non-specific barrier function, its polysaccharide group interferes with the expression of DC inflammatory factors, enhances immune tolerance, and improves intestinal stability by binding to a complex receptor on dendritic cells^56^.

Studies have shown that butyrate reduces Th17 cell production by promoting regulatory T cell (Treg) differentiation, thereby attenuating intestinal inflammatory responses^58^. Butyrate provides energy to colonocytes and reduces oxidative stress, conferring immune tolerance by triggering regulatory T cells to exert anti-inflammatory effects beyond the gastrointestinal system^59^. Several studies have indicated significant differences in the gut microflora of psoriasis patients compared to the healthy population. However, changes in specific bacterial species depend on multiple studies, which have been cross-sectional and unable to determine whether changes in the microbiota are a cause or a consequence. This study shows a potential causal relationship between the Eubacterium (coprostanoligenes group), Eubacterium (fissicatena group), and psoriasis risk.

Victivallaceae and Lachnospiraceae NK4A136 group are common gut microorganisms found in the intestines of humans and other animals^60^. They play a vital role in food digestion and absorption by breaking down complex polysaccharides, including cellulose and other indigestible plant fibres, which release beneficial nutrients like short-chain fatty acids^61^. These short-chain fatty acids provide energy to intestinal cells and promote a healthy intestinal mucosa. Additionally, they are crucial for maintaining the balance and diversity of the intestinal flora. Working with other probiotics, Victivallaceae and Lachnospiraceae NK4A136 group create a competitive advantage over harmful bacteria, effectively inhibiting their growth^62^. This balanced intestinal flora helps prevent the overgrowth of harmful bacteria and contributes to the normal functioning of the intestinal tract^63^.

Furthermore, the flora, as mentioned above plays a crucial role in regulating the immune system^64^. The interaction between the intestinal microbiota and the immune system is complex, and research has indicated that Victivallaceae and Lachnospiraceae (NK4A136 group) actively promote normal immune function. Moreover, members of the Lachnospiraceae NK4A136 group can synthesize certain nutrients that benefit the human body^65^. For instance, they can synthesize vitamin K, specific B vitamins such as folic acid and vitamin B12, which are essential for the normal physiological functioning of the body^66^. Additionally, a study demonstrated a negative correlation between Victivallaceae and Lachnospiraceae NK4A136 group and the risk of psoriasis.

Lactococcus is a common genus of lactic acid bacteria widely found in various environments, including dairy products and the intestinal tract^67^. This excellent lactose-fermenting bacteria breaks down lactose to produce lactic acid, which is particularly beneficial for individuals with lactose intolerance, as they lack the enzyme lactase required to digest lactose. By breaking down lactose, Lactococcus helps reduce lactose residue and improves lactose tolerance^68^. Moreover, Lactococcus produces several antimicrobial substances, including lactic acid, hydrogen peroxide, and lactobacilli, which effectively inhibit the growth of harmful bacteria. They compete for nutritional resources and survival space, further reducing the growth of harmful bacteria and maintaining a balanced intestinal flora^69^.

Studies have indicated that Lactococcus plays a role in regulating the immune system. It interacts with immune cells, stimulating their activity and enhancing the immune response^57^. Additionally, Lactococcus helps reduce inflammatory responses by regulating the function of the intestinal mucosal barrier^70^. The present study suggests a negative correlation between Lactococcus and the risk of psoriasis. However, further research is needed to investigate the underlying biological mechanisms linking the two^71^.

In conclusion, dysbiosis of the gut microbiota may trigger or exacerbate the development of psoriasis through various pathways, including influencing the immune system, triggering inflammatory responses, and affecting nutrient absorption and metabolism. Therefore, maintaining a healthy balance of gut microbiota is crucial for preventing and treating psoriasis. Methods such as adjusting diet, avoiding the overuse of antibiotics, and appropriately using probiotics and prebiotics may help maintain a healthy balance of gut microbiota, thus reducing the risk of occurrence and development of psoriasis^72^.

Our study has certain limitations that should be acknowledged. Firstly, we only analyzed bacterial taxa at the genus level and did not investigate more specialized levels, such as the species or strain level. This limitation may have implications for the precision of our results. Secondly, although the majority of participants in this GWAS were of European ancestry, including participants from other ethnicities might have influenced the outcomes. Consequently, the generalizability of our findings to different ethnic groups may be restricted. Thirdly, to ensure a sufficient number of instrumental variables (IVs), we selected gut microbiota IVs with a significance level of p < 1.0 × 10^–5^, which is more lenient than the traditional genome-wide significance level (p < 5 × 10^–8^). As a result, the effects of the reported bacterial traits were relatively weak, and the absence of other independent psoriasis GWAS datasets with sufficient sample sizes hindered the validation of our findings. Finally, the lack of information on psoriasis subtypes may necessitate further studies to explore this aspect once such data becomes available.

By acknowledging these limitations, we can provide a more comprehensive assessment of the study's scope and the potential impact on interpreting the results. If you have any further specific improvements or suggestions, please feel free to let me know.

## Conclusions

In summary, this study investigated the potential causal relationship between gut flora and the risk of psoriasis. The analysis revealed that specific bacterial taxa, including Mollicutes,Victivallaceae,Eubacterium (coprostanoligenes group),Eubacterium (fissicatena group), Holdemania, Lachnospiraceae (NK4A136 group), Lactococcus, and Tenericutes, were suggestively associated with the risk of psoriasis. These findings offer valuable insights into the pathogenesis of psoriasis and may open up possibilities for novel treatment approaches.

### Supplementary Information

Supplementary Table S1.

## Data Availability

The data described in this research have been sourced from two online databases, namely the MiBioGen consortium and the IEU OpenGWAS project. MiBioGen consortiumis accessible at the GCC website: https://mibiogen.gcc.rug.nl/menu/main/home/. The IEU OpenGWAS project is accessible at the GWAS Catalog website: https://gwas.mrcieu.ac.uk/. Corresponding author Feng Jiang will provide the data upon reasonable request.
