# Supplementary material for: Assessing causal relationships between gut microbiota and psoriasis: evidence from two sample Mendelian randomization analysis
Source: Sci Rep. 2024 Apr 17;14:8831. doi: 10.1038/s41598-024-59603-5 (PMC11024213; doi:10.1038/s41598-024-59603-5)
Supplement: Supplementary file 1 — Supplementary Table S1. [file 41598_2024_59603_MOESM1_ESM.docx]

**Supplementary Table S1.** Characteristics of the genetic variants associated with 8 bacterial that have been identified to be associated with the risk of psoriasis.

| Gut microbiota | SNP | Chr | Position | Effect allele | Beta | SE | *P*-value |
| --- | --- | --- | --- | --- | --- | --- | --- |
| Mollicutes | rs72901605 | 11 | 47103877 | T | -0.08419 | 0.017812 | 3.26E-06 |
| Mollicutes | rs74603314 | 14 | 46519718 | T | 0.221639 | 0.046292 | 1.56E-06 |
| Mollicutes | rs10108398 | 8 | 59440824 | G | 0.076914 | 0.015395 | 1.09E-06 |
| Mollicutes | rs11890098 | 2 | 157532549 | A | 0.074438 | 0.015339 | 9.57E-07 |
| Mollicutes | rs3768491 | 1 | 109965986 | A | -0.06811 | 0.014906 | 4.23E-06 |
| Mollicutes | rs17214486 | 14 | 97159674 | C | 0.06099 | 0.013562 | 6.61E-06 |
| Mollicutes | rs12566890 | 1 | 61850864 | T | -0.10115 | 0.023098 | 3.65E-06 |
| Mollicutes | rs78169027 | 11 | 108439087 | A | -0.10828 | 0.023729 | 5.88E-06 |
| Mollicutes | rs4885016 | 13 | 73170489 | T | -0.08196 | 0.018163 | 7.27E-06 |
| Mollicutes | rs28537087 | 15 | 95309676 | G | 0.082087 | 0.018831 | 8.07E-06 |
| Mollicutes | rs2464826 | 7 | 79490250 | A | 0.094424 | 0.021181 | 8.40E-06 |
| Mollicutes | rs6043847 | 20 | 16259524 | T | -0.11494 | 0.024861 | 4.55E-06 |
| Victivallaceae | rs67832247 | 2 | 2649472 | C | -0.12349 | 0.02667 | 3.24E-06 |
| Victivallaceae | rs34962571 | 12 | 131103256 | A | -0.18695 | 0.041841 | 6.25E-06 |
| Victivallaceae | rs4396289 | 11 | 9316730 | C | -0.15292 | 0.028853 | 1.54E-07 |
| Victivallaceae | rs2944282 | 7 | 57589803 | T | -0.1242 | 0.025703 | 1.57E-06 |
| Victivallaceae | rs11764871 | 7 | 146808977 | G | 0.127099 | 0.025656 | 7.49E-07 |
| Victivallaceae | rs6545794 | 2 | 60484909 | A | -0.19779 | 0.04105 | 5.97E-07 |
| Victivallaceae | rs2546105 | 5 | 94692439 | A | 0.126643 | 0.025711 | 1.03E-06 |
| Victivallaceae | rs7077363 | 10 | 95286954 | G | 0.148942 | 0.031837 | 2.83E-06 |
| Victivallaceae | rs62570196 | 9 | 111086170 | C | -0.2461 | 0.048308 | 2.7E-07 |
| Victivallaceae | rs7860510 | 9 | 94128796 | G | 0.164855 | 0.037513 | 9.6E-06 |
| Victivallaceae | rs7627405 | 3 | 9968555 | C | -0.13416 | 0.030143 | 8.19E-06 |
| Victivallaceae | rs61702987 | 2 | 31211657 | T | 0.145501 | 0.030001 | 3.08E-06 |
| Victivallaceae | rs7314815 | 12 | 102525047 | G | 0.101088 | 0.022559 | 6.4E-06 |
| Victivallaceae | rs11671100 | 19 | 711637 | A | -0.16003 | 0.034947 | 4.08E-06 |
| Eubacteriumcoprostanoligenesgroup | rs17159861 | 7 | 31085162 | C | 0.096223 | 0.016839 | 1.04E-08 |
| Eubacteriumcoprostanoligenesgroup | rs9648214 | 7 | 16380806 | T | -0.08287 | 0.016431 | 2.52E-07 |
| Eubacteriumcoprostanoligenesgroup | rs115325767 | 1 | 200638489 | G | 0.078332 | 0.017472 | 2.62E-06 |
| Eubacteriumcoprostanoligenesgroup | rs12906958 | 15 | 36911598 | C | -0.05332 | 0.011593 | 4.35E-06 |
| Eubacteriumcoprostanoligenesgroup | rs4076415 | 15 | 86440997 | T | 0.051515 | 0.011029 | 1.99E-06 |
| Eubacteriumcoprostanoligenesgroup | rs6762473 | 3 | 127139574 | C | 0.052158 | 0.011235 | 4.26E-06 |
| Eubacteriumcoprostanoligenesgroup | rs10444197 | 10 | 2215931 | A | -0.05058 | 0.011346 | 5.98E-06 |
| Eubacteriumcoprostanoligenesgroup | rs62024432 | 15 | 97869293 | C | -0.07697 | 0.01721 | 7.5E-06 |
| Eubacteriumcoprostanoligenesgroup | rs2644213 | 10 | 84506096 | G | 0.053885 | 0.012124 | 9.86E-06 |
| Eubacteriumcoprostanoligenesgroup | rs11720857 | 3 | 113794507 | C | 0.063078 | 0.014448 | 9.26E-06 |
| Eubacteriumcoprostanoligenesgroup | rs4717831 | 7 | 73306506 | A | 0.078678 | 0.017404 | 9.18E-06 |
| Eubacteriumcoprostanoligenesgroup | rs1020520 | 7 | 33603146 | T | -0.05907 | 0.013293 | 8.89E-06 |
| Eubacteriumcoprostanoligenesgroup | rs76898927 | 3 | 81595477 | G | 0.123055 | 0.026637 | 4.79E-06 |
| Eubacteriumcoprostanoligenesgroup | rs11052069 | 12 | 32713919 | T | 0.047783 | 0.010783 | 9.38E-06 |
| Eubacteriumcoprostanoligenesgroup | rs79895140 | 2 | 50797401 | T | -0.0641 | 0.014115 | 8.62E-06 |
| Eubacteriumfissicatenagroup | rs3771393 | 2 | 71145246 | C | 0.130842 | 0.026667 | 7.38E-07 |
| Eubacteriumfissicatenagroup | rs2733072 | 8 | 5433699 | G | 0.109644 | 0.022831 | 1.49E-06 |
| Eubacteriumfissicatenagroup | rs7104872 | 11 | 115165111 | G | 0.138612 | 0.029191 | 2.73E-06 |
| Eubacteriumfissicatenagroup | rs151257695 | 7 | 73043561 | A | 0.20951 | 0.045485 | 3.1E-06 |
| Eubacteriumfissicatenagroup | rs11876297 | 18 | 45753272 | T | 0.131469 | 0.028171 | 2.67E-06 |
| Eubacteriumfissicatenagroup | rs6934739 | 6 | 39940437 | A | 0.111463 | 0.025279 | 9.75E-06 |
| Eubacteriumfissicatenagroup | rs10147907 | 14 | 89484373 | T | 0.172263 | 0.039601 | 8.27E-06 |
| Eubacteriumfissicatenagroup | rs1768152 | 3 | 39604954 | T | 0.139489 | 0.031619 | 8.7E-06 |
| Eubacteriumfissicatenagroup | rs11818408 | 10 | 96758467 | G | 0.10585 | 0.023711 | 8.2E-06 |
| Holdemania | rs6133067 | 20 | 3839754 | T | 0.091083 | 0.017856 | 5.17E-07 |
| Holdemania | rs1867876 | 11 | 18768068 | T | 0.084292 | 0.016219 | 2.74E-07 |
| Holdemania | rs9500080 | 6 | 105737322 | C | 0.092676 | 0.017889 | 4.09E-07 |
| Holdemania | rs150096134 | 12 | 39589195 | T | 0.162139 | 0.033209 | 2.38E-06 |
| Holdemania | rs41438744 | 14 | 102553414 | C | -0.12487 | 0.027013 | 2.44E-06 |
| Holdemania | rs77293403 | 5 | 77388406 | A | 0.164556 | 0.034177 | 1.77E-06 |
| Holdemania | rs80149660 | 10 | 129908282 | C | -0.23299 | 0.051918 | 6.04E-06 |
| Holdemania | rs116500994 | 13 | 81685543 | G | -0.13755 | 0.029339 | 2.34E-06 |
| Holdemania | rs55888180 | 17 | 76443497 | C | 0.128671 | 0.028279 | 5.89E-06 |
| Holdemania | rs4146507 | 5 | 106209093 | C | 0.079488 | 0.0177 | 7.23E-06 |
| Holdemania | rs111745969 | 15 | 93110222 | A | 0.120677 | 0.026578 | 3.71E-06 |
| Holdemania | rs113593397 | 8 | 123973840 | A | -0.12893 | 0.028253 | 9.36E-06 |
| Holdemania | rs9529719 | 13 | 70856516 | T | 0.074038 | 0.016049 | 5.97E-06 |
| Holdemania | rs10885477 | 10 | 115332413 | T | -0.13514 | 0.030189 | 8.6E-06 |
| Holdemania | rs967319 | 3 | 60615680 | T | 0.078864 | 0.017674 | 8.38E-06 |
| Holdemania | rs12701617 | 7 | 38292857 | A | -0.06606 | 0.014943 | 9.52E-06 |
| Holdemania | rs73139538 | 7 | 63323145 | G | -0.14859 | 0.032747 | 7.77E-06 |
| Holdemania | rs11080063 | 17 | 26789271 | G | -0.06652 | 0.014984 | 6.67E-06 |
| LachnospiraceaeNK4A136group | rs12362320 | 11 | 3922055 | G | 0.057317 | 0.011547 | 8.04E-07 |
| LachnospiraceaeNK4A137group | rs954878 | 1 | 54578401 | A | -0.05207 | 0.010908 | 1.78E-06 |
| LachnospiraceaeNK4A138group | rs7832116 | 8 | 4842929 | A | -0.07148 | 0.01517 | 3.57E-06 |
| LachnospiraceaeNK4A139group | rs7616165 | 3 | 190415145 | G | -0.23054 | 0.048346 | 2.77E-06 |
| LachnospiraceaeNK4A140group | rs76193507 | 3 | 161749981 | A | -0.22973 | 0.049978 | 2.93E-06 |
| LachnospiraceaeNK4A141group | rs11263806 | 17 | 35240097 | A | -0.05246 | 0.011676 | 5.07E-06 |
| LachnospiraceaeNK4A142group | rs73044693 | 19 | 51256120 | A | -0.10758 | 0.022988 | 3.57E-06 |
| LachnospiraceaeNK4A143group | rs160061 | 5 | 6116659 | A | 0.051383 | 0.010809 | 2.12E-06 |
| LachnospiraceaeNK4A144group | rs7073658 | 10 | 62175024 | T | -0.04996 | 0.010969 | 5.27E-06 |
| LachnospiraceaeNK4A145group | rs68104925 | 14 | 100177681 | T | -0.05491 | 0.011538 | 2.37E-06 |
| LachnospiraceaeNK4A146group | rs12611395 | 19 | 21806125 | A | -0.09025 | 0.019965 | 5.83E-06 |
| LachnospiraceaeNK4A147group | rs2880566 | 17 | 30016653 | T | 0.059958 | 0.013469 | 5.61E-06 |
| LachnospiraceaeNK4A148group | rs28540839 | 8 | 84735080 | A | 0.050828 | 0.011059 | 9.34E-06 |
| LachnospiraceaeNK4A149group | rs59805249 | 5 | 90289997 | T | 0.093616 | 0.020798 | 9.45E-06 |
| LachnospiraceaeNK4A150group | rs10952110 | 7 | 8497922 | G | 0.048771 | 0.010961 | 9.08E-06 |
| LachnospiraceaeNK4A151group | rs4955932 | 3 | 55181375 | T | -0.04923 | 0.010939 | 7.05E-06 |
| Lactococcus | rs55910161 | 10 | 71518643 | C | 0.146426 | 0.030737 | 2.36E-06 |
| Lactococcus | rs12621813 | 2 | 31266049 | G | 0.108423 | 0.023998 | 6.61E-06 |
| Lactococcus | rs2293361 | 2 | 54114864 | C | -0.19922 | 0.043097 | 1.4E-06 |
| Lactococcus | rs7992246 | 13 | 113472525 | T | 0.104232 | 0.023079 | 4.45E-06 |
| Lactococcus | rs34757988 | 18 | 64699550 | G | 0.122294 | 0.022898 | 8.95E-08 |
| Lactococcus | rs757872 | 22 | 31532568 | G | 0.140799 | 0.027582 | 4.37E-07 |
| Lactococcus | rs123059 | 17 | 2699935 | T | -0.13671 | 0.027469 | 1.27E-06 |
| Lactococcus | rs4766997 | 12 | 113161438 | C | 0.114599 | 0.023839 | 2.06E-06 |
| Lactococcus | rs10417872 | 19 | 28767353 | T | 0.118306 | 0.024522 | 1.29E-06 |
| Lactococcus | rs6674304 | 1 | 116887742 | C | 0.200758 | 0.044212 | 6.18E-06 |
| Lactococcus | rs17168302 | 7 | 14650221 | G | 0.191857 | 0.042476 | 6.29E-06 |
| Tenericutes | rs74603314 | 14 | 46519718 | T | 0.221639 | 0.046292 | 1.56E-06 |
| Tenericutes | rs10108398 | 8 | 59440824 | G | 0.076914 | 0.015395 | 1.09E-06 |
| Tenericutes | rs11890098 | 2 | 157532549 | A | 0.074438 | 0.015339 | 9.57E-07 |
| Tenericutes | rs72901605 | 11 | 47103877 | T | -0.08419 | 0.017812 | 3.26E-06 |
| Tenericutes | rs3768491 | 1 | 109965986 | A | -0.06811 | 0.014906 | 4.23E-06 |
| Tenericutes | rs17214486 | 14 | 97159674 | C | 0.06099 | 0.013562 | 6.61E-06 |
| Tenericutes | rs12566890 | 1 | 61850864 | T | -0.10115 | 0.023098 | 3.65E-06 |
| Tenericutes | rs78169027 | 11 | 108439087 | A | -0.10828 | 0.023729 | 5.88E-06 |
| Tenericutes | rs28537087 | 15 | 95309676 | G | 0.082087 | 0.018831 | 8.07E-06 |
| Tenericutes | rs4885016 | 13 | 73170489 | T | -0.08196 | 0.018163 | 7.27E-06 |
| Tenericutes | rs2464826 | 7 | 79490250 | A | 0.094424 | 0.021181 | 8.4E-06 |
| Tenericutes | rs6043847 | 20 | 16259524 | T | -0.11494 | 0.024861 | 4.55E-06 |
|  |  |  |  |  |  |  |  |
